# Case report: Identification of a novel variant p.Gly215Arg in the *CHN1* gene causing Moebius syndrome

**DOI:** 10.3389/fgene.2024.1291063

**Published:** 2024-01-31

**Authors:** Carmen Manso-Bazús, Nino Spataro, Elisabeth Gabau, Viviana P. Beltrán-Salazar, Juan Pablo Trujillo-Quintero, Nuria Capdevila, Anna Brunet-Vega, Neus Baena, A Arockia Jeyaprakash, Victor Martinez-Glez, Anna Ruiz

**Affiliations:** ^1^ Center for Genomic Medicine, Parc Taulí Hospital Universitari, Institut d’Investigació i Innovació Parc Taulí (I3PT-CERCA), Universitat Autònoma de Barcelona, Sabadell, Spain; ^2^ Paediatric Service, Parc Taulí Hospital Universitari, Institut d’Investigació i Innovació Parc Taulí (I3PT-CERCA), Universitat Autònoma de Barcelona, Sabadell, Spain; ^3^ Radiology Service, Parc Taulí Hospital Universitari, Institut d’investigación i innovació Parc Taulí (I3PT-CERCA), Universitat Autónoma de Barcelona, Sabadell, Spain; ^4^ Wellcome Centre for Cell Biology, University of Edinburgh, Edinburgh, United Kingdom; ^5^ The Gene Centre and Department of Biochemistry, Ludwig Maximilian Universität, München, Germany

**Keywords:** moebius syndrome, genetic diagnosis, CHN1, novel variant, congenital dysinnervation syndromes

## Abstract

**Background:** Moebius Syndrome (MBS) is a rare congenital neurological disorder characterized by paralysis of facial nerves, impairment of ocular abduction and other variable abnormalities. MBS has been attributed to both environmental and genetic factors as potential causes. Until now only two genes, *PLXND1* and *REV3L* have been identified to cause MBS.

**Results:** We present a 9-year-old male clinically diagnosed with MBS, presenting facial palsy, altered ocular mobility, microglossia, dental anomalies and congenital torticollis. Radiologically, he lacks both abducens nerves and shows altered symmetry of both facial and vestibulocochlear nerves. Whole-exome sequence identified a *de novo* missense variant c.643G>A; p.Gly215Arg in *CHN1*, encoding the α2-chimaerin protein. The p.Gly215Arg variant is located in the C1 domain of CHN1 where other pathogenic gain of function variants have been reported. Bioinformatic analysis and molecular structural modelling predict a deleterious effect of the missense variant on the protein function.

**Conclusion:** Our findings support that pathogenic variants in the *CHN1* gene may be responsible for different cranial congenital dysinnervation syndromes, including Moebius and Duane retraction syndromes. We propose to include *CHN1* in the genetic diagnoses of MBS.

## Introduction

Moebius syndrome (MBS) is a rare congenital neurological disease characterized by non-progressive facial palsy and impairment of ocular abduction, due to uni or bilateral paralysis or weakness of the facial (VII) and abducens (VI) cranial nerves. It can also be associated to paralysis of other cranial nerves (most notably cranial nerves V, IX, X, and XII). Other abnormalities include lingual hypoplasia, sensorineural hearing loss, craniofacial malformations (epicanthic folds, micrognathia), and abnormalities of the extremities (syndactyly, pes planus, valgus femur) (Terzis and Noah, 2002). Face and mouth functional anomalies implicate facial weakness, difficulties in speaking, eating, sucking and swallowing.

MBS is classified as a congenital cranial dysinnervation disorder (CCDD). These are disorders caused by developmental abnormalities of cranial nerves/nuclei resulting in primary or secondary dysinnervation. The CCDDs include Duane retraction syndrome (DRS), congenital fibrosis of the external ocular muscles (CFEOM), hereditary congenital facial palsy (HCFP), horizontal gaze palsy with progressive scoliosis (HGPPS) and MBS (Gutowski and Chilton, 2015; Oystreck, 2018; Jia et al., 2022).

The prevalence of MBS is estimated to be 1 in 10,000 to 1 in 2,500 live births with equal incidence in both sexes. Most patients present normal intelligence, while rare cases of autistic-like behaviours (0%–5%) and mild intellectual disability (9%–15%) have been reported (Picciolini et al., 2016).

Since the initial descriptions by von Graefe in 1880 (von Graefe, 1880) and by Moebius in 1888 (Möbius, 2008) it has been debated whether MBS has a genetic or a non-genetic aetiology. Most patients have a sporadic occurrence with a limited number of atypical familial cases (Tischfield et al., 2005; Schröder et al., 2013). Both intrauterine environmental factors and genetic causes have been proposed. Prenatal exposure to misoprostol or cocaine can lead to disruption of blood vessel migration during development and hindbrain hypoxia, resulting in cranial nerve dysfunction.

In 2015, *de novo* pathogenic variants affecting two genes, *PLXND1* and *REV3L* were reported in six unrelated sporadic Moebius patients from a cohort of 110 patients. *PLXND1* and *REV3L* are both involved in hindbrain development. Analysis of *Plxnd1* and *Rev3l* mutant mice shows that disruption of these genes converge at the facial branchiomotor nucleus, affecting either motoneuron migration or proliferation (Tomas-Roca et al., 2015). The low frequency of pathogenic variants detected in these two genes as a cause of MBS suggests that *de novo* mutations in other genes may be responsible for MBS.

Here, we identified, using whole exome sequencing, a *de novo* novel heterozygous variant in the *CHN1* gene in a patient diagnosed with MBS. Gain of function variants in the *CHN1* gene have been previously shown to cause DRS, which is a congenital eye movement disorder characterised by variable horizontal duction deficits, with palpebral fissure narrowing and globe retraction on attempted adduction.

## Material and methods

### Patients and subjects

Patient genomic DNA was extracted from peripheral blood leukocytes from the patient and patient’s parents. Written informed consent was obtained from the patient’s parents.

### Exome sequencing and data analysis

Exome capture was performed using the KAPA HyperExome probes (Roche) and sequenced on an Illumina Novaseq platform (Illumina, San Diego, CA, United States) at the National Centre of Genomic Analysis (CNAG-CRG, Barcelona, Spain), producing 2 × 150 nucleotides paired-end reads. Bioinformatics analysis was performed as described in Spataro et al., 2023. Sanger direct sequencing of candidate variants was done in the patient and parents to determine the inheritance pattern. Finally, variants were classified following the American College of Medical Genetics and Genomics and the Association for Molecular Pathology (ACMG/AMP) guidelines (Richards et al., 2015) and the recommendations provided by the Sequence Variant Interpretation working group at ClinGen (https://clinicalgenome.org/working-groups/sequence-variant-interpretation/).

### Magnetic resonance imaging

Magnetic Resonance (MR) examination was performed using a 1.5 T Magnetic Resonance Imaging (MRI) scanner (Aera; Siemens Medical Solutions, Erlangen, Germany).

### Structural modelling of the CHN1 p. Gly215Arg missense variant

The p. Gly215Arg variant was modelled using the published CHN1 crystal structure (3CXL) as a template using PyMOL, a molecular graphics program (The PyMOL Molecular Graphics System, Version 1.2r3pre, Schrödinger, LLC).

## Results

### Case clinical description

The proband is a 9-year-old male clinically diagnosed with MBS, son of healthy non-consanguineous parents.

**FIGURE 1:**
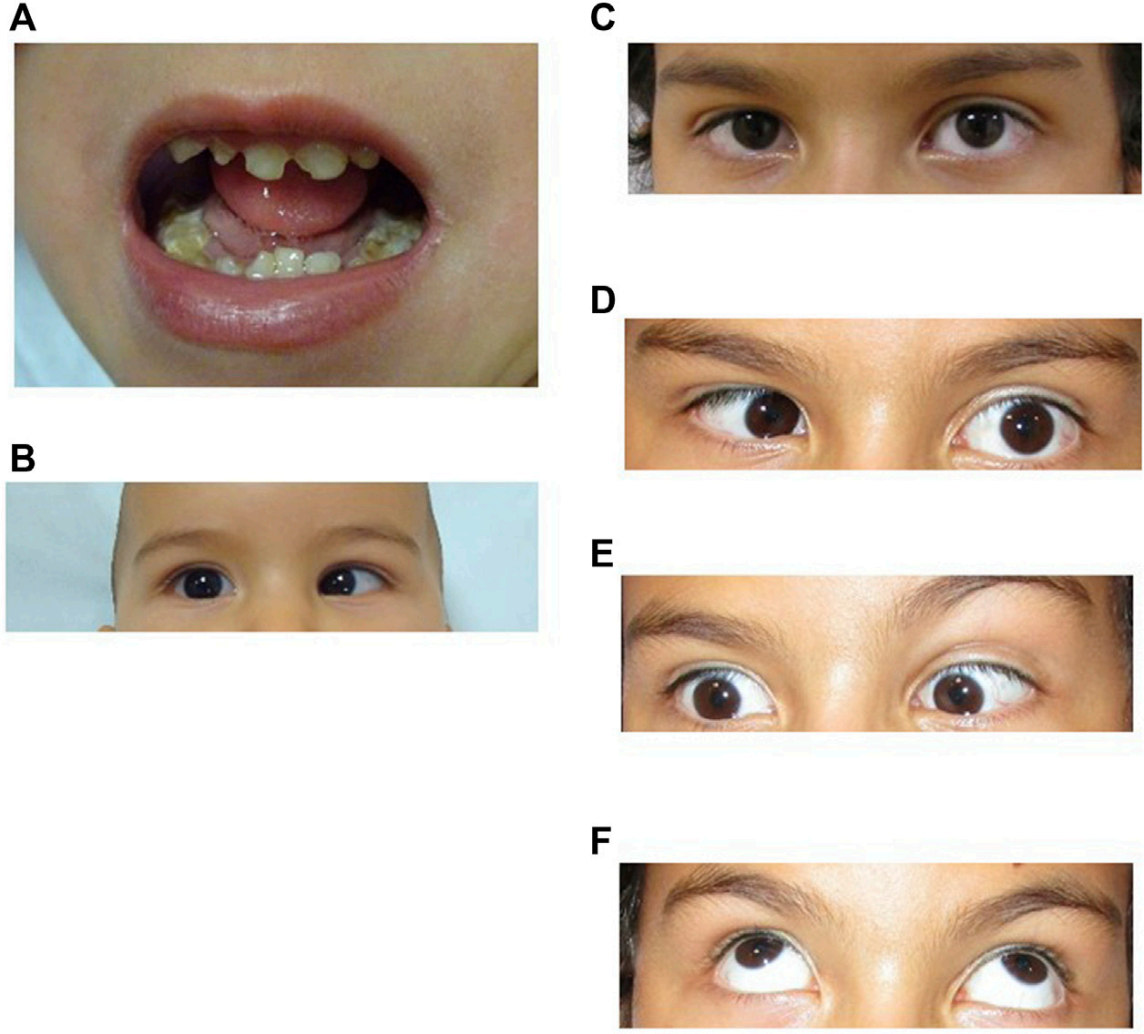
**(A)**, Patient at 3 years 10 month old. Microglossia and dental anomalies. **(B)**, Patient at 10 months of age showing limited outward gaze (abduction) of the right eye. **(C–F)**. Patient at 9 years old. Ocular motility patterns. **(C)**, Straight gaze showing mild esotropia of the right eye. **(D)**, Horizontal left gaze showing limited abduction on the left eye. **(E)**, Horizontal right gaze showing limited abduction on the right eye. **(F)**, Full vertical eye movement. Exotropia in upper gaze.

He was born at term after uneventful pregnancy and delivery. After birth, he had significant difficulties in sucking, presenting microglossia, and remaining in hospital for a month. He was referred to a clinical genetics service at 2 months of age due to the microglossia (Figure 1A), limited tongue movements, left-winged scapula, and altered ocular mobility (Figure 1B). He presented bilateral convergent strabismus, bilateral abduction limitation and congenital torticollis (face to the right). He presented significant feeding and swallowing difficulties during his first 6 months, which improved later in life. Lack of facial expression was detected before 1 year of age.

At present, his cranial circumference, weight and height are within the normal range. He has been operated twice on both eyes to correct convergent strabismus (2.5 and 6 years) improving the torticollis. His left eye position has been centred but remains with limited abduction and his right eye remains with limited abduction and mild esotropia (Figures 1C–E). There is no limitation in his upper gaze (Figure 1F).

**FIGURE 2:**
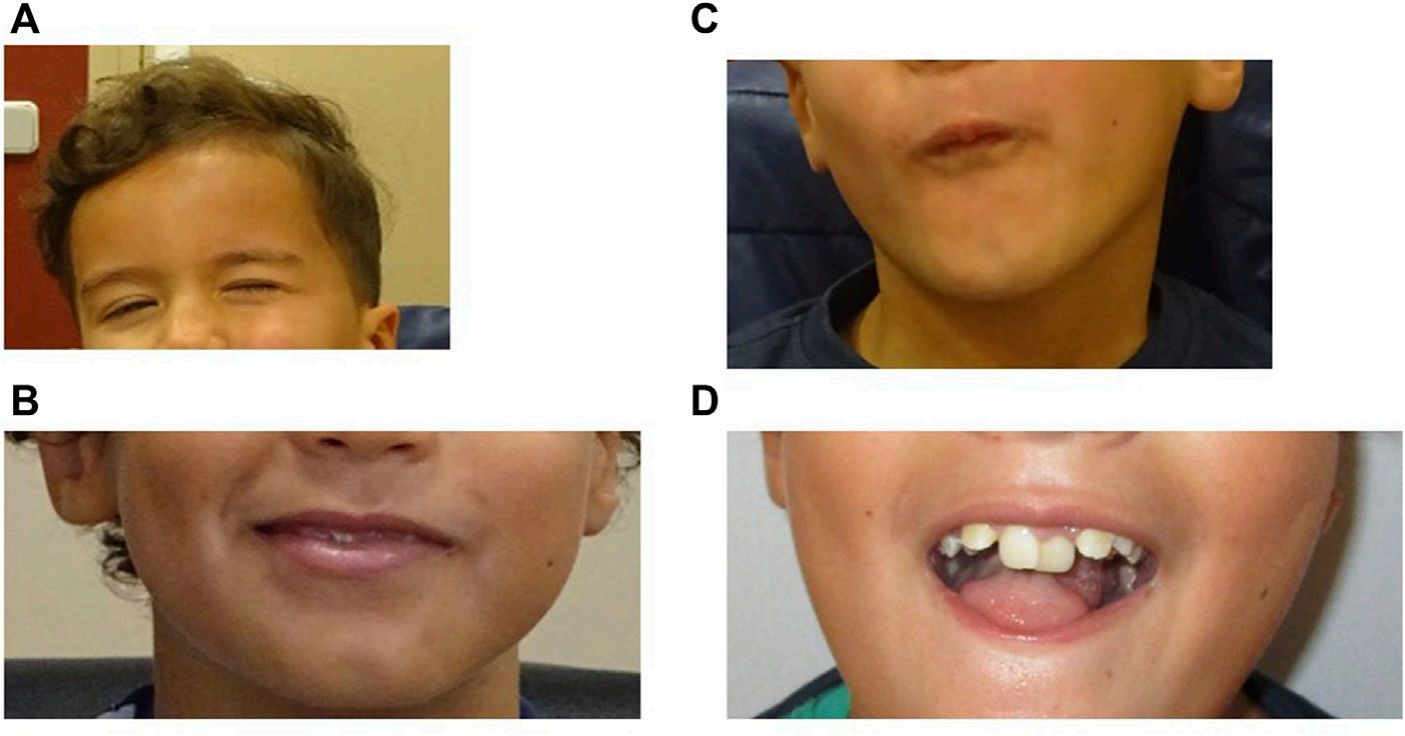
**(A)**, Patient at 4 years 10 month old. Movements of the lower part of the forehead are reduced. **(B,C)**, Patient at 9 years old showing mild facial palsy. **(B)**, the patient cannot fully smile and when he smiles there is a mild asymmetry. **(C)**, Limited mouth movements (he cannot blow). **(D)**, Patient at 9 years old. Microglossia and dental anomalies.

He presents hypomimia with reduced movements of the lower part of the forehead (Figure 2A). He also presents mild facial palsy and limited mouth movements. He cannot fully smile and when he smiles there is a mild asymmetry (Figure 2B) and he cannot blow (Figure 2C). According to family reports and medical health record, his facial expression has improved over the years. He still has a marked microglossia with altered movements, which limits language articulation (not swallowing). He presents malposition of the teeth (requiring orthodontics) and alterations of the dental enamel (presenting multiple cavities and needing corrective interventions since early childhood) (Figure 2D). Finally, his left winged scapula persists limiting abduction, with atrophy of the supraspinatus and dorsalis muscle on the same side.

Psychomotor development showed mild delay in the main items (crawling at around 14 months and autonomous walking at 20 months, due to hypotonia). Language development was slow because of difficulties in articulation, due to microglossia, which persist today. The patient showed no problems with receptive language. Social and learning development is within the normal range, with mild attentional symptoms that do not meet the diagnostic criteria for ADHD. Cognitive capacity is normal and there are no difficulties in acquiring basic and instrumental daily activities (such as dressing, eating, personal hygiene, transportation).

### Imaging analysis

**FIGURE 3:**
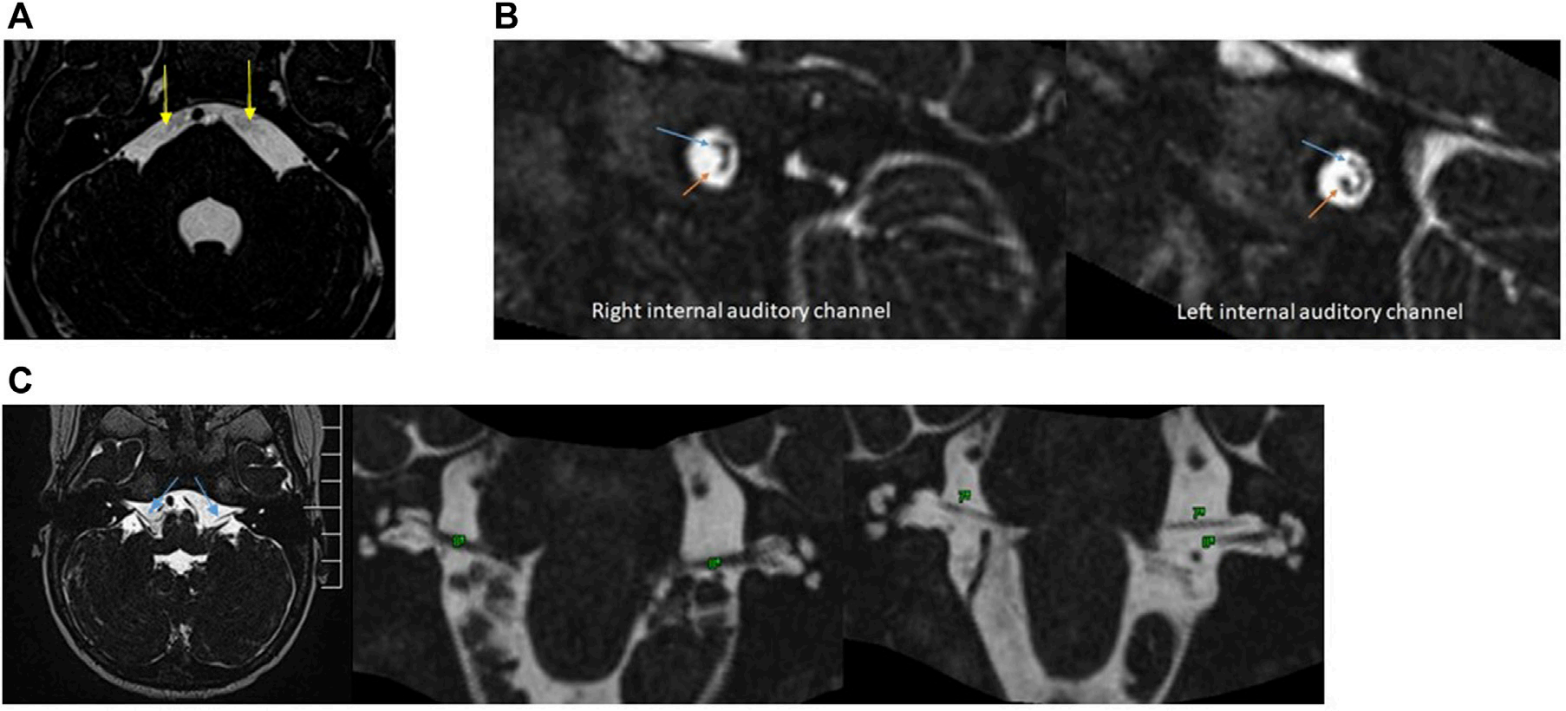
**(A)**, 3D-constructive interference in steady State (CISS) sequences. Note the absence of bilateral VI nerves (yellow arrows). **(B)**, 3D-CISS sequences with reconstructions perpendicular to the bilateral internal auditory channel show an asymmetry in the position of VII (blue arrows) and VIII nerves (orange arrows). The left side is normal. **(C)**, 3D-CISS sequences in the axial plane with reconstructions in the coronal plane demonstrate the presence of a cisternal segment of bilateral facial (VII) (blue arrows) and vestibulocochlear nerve (VIII).

Radiologically, MS is characterised by bilateral absent or hypoplasic cisternal and canalicular portion of the facial nerve, and cisternal portion of the abducens nerve (Razek et al., 2021). Patient’s MRI performed at 1 year of age did not identify the intracanicular and cisternal segments of the abducens nerves (VI) and showed an asymmetry in the normal position of the right VII and VIII nerves into the internal auditory channel (Figures 3A,B). However, the cisternal segment of bilateral facial nerve was present (Figure 3C). There was also microglossia (as seen clinically), but it was not possible to evaluate the lower cranial nerves. The trigeminal nerve was normal.

### Genetic analysis

**FIGURE 4:**
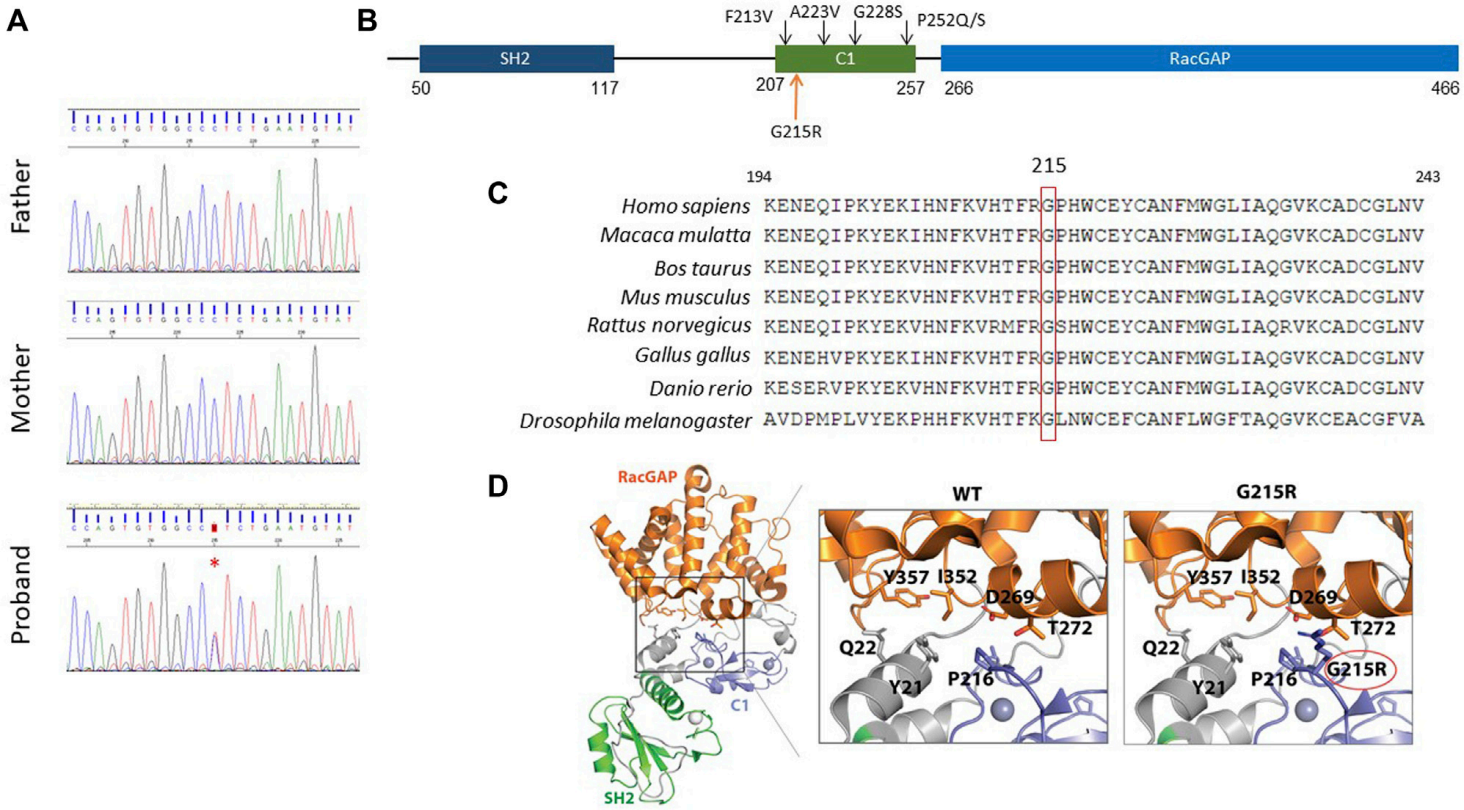
Molecular characterisation of the c.643G>A; p. Gly215Arg variant. **(A)**, *De novo* inheritance pattern of *CHN1* c.643G>A variant. A red asterisk indicates the variant. **(B)**, Schematic structure of the human CHN1 protein. The upper black arrows indicate previously reported pathogenic variants in the C1 domain. **(C)**, Multiple-sequence alignment showing the conservation of Gly215 residue in CHN1 across evolution. **(D)**, Cartoon representation of the protein structure CHN1. Insets show close-up views of (left) the interaction mediated by CHN1 Gly215 with nearby amino acid residues in stick representation.

Using whole-exome sequencing (WES) a novel missense variant c.643G>A was detected in the *CHN1* gene (NM_001822.7), which encodes the RacGAP signalling protein α2-chimaerin. The variant was confirmed by Sanger sequencing in the patient and was absent in both parents indicating that it has raised *de novo* (Figure 4A).

This c.643G>A variant replaces a glycine residue for an arginine in the C1 domain of the protein (p.Gly215Arg) (Figure 4B). The amino acid sequence alignment analysis indicated that Gly215 in the CHN1 protein is highly conserved throughout evolution (Figure 4C). The p.Gly215Arg variant is considered damaging or potentially disease-causing by most *in silico* predictors used (Supplementary Table S1) and is not present in the population databases gnomAD (Karczewski et al., 2020). In addition, prediction of protein stability determined by different software showed protein destabilisation indicated by a decrease in Gibbs free energy (Supplementary Table S2).

The α2-chimaerin longest isoform has an N-terminal SH2 domain (50-117aa), a C1 domain (207-255aa) that binds to diacylglycerol and a RacGAP domain (266-456aa) that interacts and downregulates Rac activity. α2-chimaerin is located in the cytoplasm in an inactive closed conformation. It unfolds and translocates to the membrane in response to DAG signalling, exposing the RacGAP domain and inactivating Rac. Previously identified variants have been shown to hyper-activate α2-chimaerin RacGAP activity either by destabilizing its closed conformation or by directly altering DAG or Rac binding (Miyake et al., 2008).

Gly 215 is located within the C1 domain in a loop that forms the interaction interface with the Rac GAP domain. Structural modelling of the p.Gly215Arg substitution showed steric clashes between amino acid Arg215 and RacGAP domain residues Thr272 and Asp269. This suggests that the p.Gly215Arg might alter the interface between the C1 and the RacGAP binding domain, changing their relative orientation and leading to an ‘open’ conformation, facilitating its translocation to the membrane (Figure 4D).

The c.643G>A (p. Gly215Arg) in the *CHN1* gene was classified as likely pathogenic according to the American College of Medical Genetics (ACMG) guidelines, meeting the following criteria: 1) PM1, located in a functional domain of the protein; 2) PM6, *de novo*; 3) PM2_supporting, absent in population databases; 4) PP2; Missense variant in a gene with low rate of benign missense mutations and for which missense mutation is a common mechanism of a disease and 5) PP3, computational evidence shows a deleterious effect. The variant has been submitted to the ClinVar (Landrum et al., 2018) database (ref. VCV001708248.1).

## Discussion

Pathogenic variants in the *CHN1* gene have been mostly associated with Duane retraction syndrome (Miyake et al., 2008). Although Duane syndrome is commonly a sporadic disorder, 10% of cases are familial inherited cases. At least two other genes have been associated to Duane syndrome: *MAFB* (Park et al., 2016) and *SALL4* (Al-Baradie et al., 2002).

Here, we describe a patient diagnosed with Moebius syndrome, carrier of a novel *de novo* missense variant in the *CHN1* gene. *CHN1* encodes the signalling protein alpha2-chimaerin, a crucial regulator of axon guidance in the ocular motor system (Miyake et al., 2008; Chilton and Guthrie, 2017). Pathogenic variants in the *CHN1* gene have been shown to hyperactivate a2-chimerin RacGAP activity by either destabilizing the protein inactive closed conformation or by directly altering DAG or Rac binding resulting in aberrant cranial motor neuron development (Miyake et al., 2008). Moreover, both heterozygous and homozygous knockin mice harbouring a Duane syndrome gain-of-function missense mutation show eye movement abnormalities and unilateral or bilateral globe retraction. α2-chimaerin mutation alter the primary development of the abducens, trochlear, and C1 nerves. Mechanistically, these three different motor neuron populations use ephrin/EphA4-mediated signaling pathways upstream of mutant α2-chimaerin in distinct manners to guide developing axons (Nugent et al., 2017).

Bioinformatic analysis and structural modelling of the p.Gly215Arg variant supports a putative gain of function effect, where the p.Gly215Arg substitution will result in the destabilization of the closed conformation of the CHN1 protein, increasing its RacGAP activity as has been observed for other variants in the C1 domain of the *CHN1* gene. Variants located in the C1 domain such as Phe213Val, Ala223Val, and Pro252/Gln/Ser have been shown to enhance Rac-GAP activity by destabilizing the closed conformation of α2-chimaerin and enhancing membrane translocation (Miyake et al., 2008).

Here we show that the novel p.Gly215Arg variant in the *CHN1* gene is associated with MBS. The patient presents facial palsy, altered ocular mobility, microglossia and congenital torticollis. Radiologically, he lacks both abducens nerves and shows altered symmetry of both facial and vestibulocochlear nerves. Variants in *CHN1* have been postulated to be mainly associated with bilateral DRS, often with some abnormalities in vertical gaze and other rare somatic disturbances (Miyake et al., 2011). However, a recent report describes a missense variant in the *CHN1* gene (p.Tyr221His) in a child and his father with DRS, associated with swallowing difficulties and unilateral trapeze aplasia (Angelini et al., 2021). All symptoms are related to anomalies in different cranial nerves: VI, IX, X, XI, and XII suggesting that variants in the *CHN1* gene affect the development of other cranial nerves in addition to the oculomotor system (Angelini et al., 2021). It has been shown that α2-chimaerin is expressed in all developing cranial motor neurons, including the abducens, trochlear and oculomotor, as well as in most developing neurons throughout the central and peripheral nervous system. Our data support that the gain of function variants in the *CHN1* gene may be responsible for a spectrum of phenotypes, from DRS to MBS, affecting the normal development of cranial motor neurons. Further analyses are needed to show whether variants in *CHN1* may cause other congenital cranial dysinnervation syndromes and to characterize their degree of variability in clinical expressivity.

To date, only two genes have been proposed to cause MBS (*PLXND1* and *REV3L*). Animal models indicate that *PLXND1* and *REV3L* cause hypoplasia of the facial motor nucleus while *CHN1* knock-in mice present alteration in the primary development of the abducens, trochlear, and C1 nerves (Tomas-Roca et al., 2015; Nugent et al., 2017). Apparently, the three genes act by different molecular pathways converging in defects of motoneuron migration, proliferation and axon guidance (Tomas-Roca et al., 2015; Nugent et al., 2017).

We propose that *CHN1* should be included in the genetic diagnoses of MBS as well as other CCDDs. CCDDs represent a group of developmental disorders that commonly involve disturbances to ocular motility. There is phenotypic variability between CCDDs subtypes with overlap between entities. Some conditions can be distinguished by the presence of other associated features. For instance, HGPPS individuals are expected to have scoliosis while patients with MBS have facial weakness. While it can be difficult to a clinician to distinguish the type and degree of the ocular phenotype, genetic diagnosis may contribute to diagnosis confirmation.

## Data Availability

The datasets for this article are not publicly available due to concerns regarding participant/patient anonymity. Requests to access the datasets should be directed to the corresponding author.
