# Supplementary material for: Case report: Identification of a novel variant p.Gly215Arg in the CHN1 gene causing Moebius syndrome
Source: Front Genet. 2024 Jan 31;15:1291063. doi: 10.3389/fgene.2024.1291063 (PMC10865368; doi:10.3389/fgene.2024.1291063)
Supplement: Supplementary file 1 [file Table1.DOCX]

Supplementary Table 1 . *In silico* analysis of the p.Gly215Arg variant using bioinformatics predictors.

| In silico tool | Prediction | Score |
| --- | --- | --- |
| PROVEAN | Deleterious | -7.37 |
| PolyPhen-2 | Probably Damaging | 1 |
| MutPred | Uncertain | 0.588 |
| CADD | Deleterious | 32 |
| PrimateAI | Damaging | 0.916 |
| REVEL | Pathogenic | 0.792 |
