# Supplementary material for: Case report: Identification of a novel variant p.Gly215Arg in the CHN1 gene causing Moebius syndrome
Source: Front Genet. 2024 Jan 31;15:1291063. doi: 10.3389/fgene.2024.1291063 (PMC10865368; doi:10.3389/fgene.2024.1291063)
Supplement: Supplementary file 2 [file Table2.DOCX]

Supplementary Table 2. Prediction of protein stability changes due to p.Gly215Arg aminoacid substitution using various softwares.

| Software | ΔΔG value | Prediction |
| --- | --- | --- |
| mCSM | -0,695 Kcal/mol | Destabilizing |
| DUET | -0,757 Kcal/mol | Destabilizing |
| SDM | -2,44 Kcal/mol | Destabilizing |
| I-Mutant 2.0 | -2,48 Kcal/mol | Destabilizing |
